# Financial institutions digital transformation: the stages of the journey and business metrics to follow

**DOI:** 10.1057/s41264-023-00223-x

**Published:** 2023-04-10

**Authors:** Aristides Papathomas, George Konteos

**Affiliations:** 1 Department of Business Administration, University of Western Macedonia, Macedonia, Greece; 2 Department of Business Administration, School of Economics, University of Western Macedonia, Macedonia, Greece

**Keywords:** Digital transformation, Digital banking, Incumbent banks, Metrics, Key performance indicator, Key tracking indicators

## Abstract

This paper examines the stages of the digital transformational path that lies in front of incumbent banks in their conversion into digitally driven institutions and contributes by providing clarity in the parameters that define each stage and the key metrics to be tracked. It is a general review paper, with main tools employed the relevant scholar and grey literature & field observations. The paper identifies three phases for banking institutions’ digital transformation and proceeds with defining the characteristics of the phases and the distinct actions required for an institution to progress through them, employing a set of proposed key tracking indicators. The outcome adds to the, rather limited, academic literature on the subject and can be applied to all relevant banking institutions. Research needs further insides to articulate better the findings and expand them on a cross-examination of relevant theories and approaches. This paper aims at contributing to a growing, contemporary discussion, hopefully assisting in greater collaboration between practitioners and academics.

## Introduction

Digital transformation is an ever-increasing popular trend that cannot be ignored by any individual, organization or country. Technological advances like Internet of Things (IoT), artificial intelligence (AI), automation, remote monitoring, predictive maintenance, additive manufacturing, big data, cloud computing, analytics are seen as new, disruptive market opportunities for firms (Parida et al 2019; Warner and Wäger, 2019, Ferreira et al 2019). Most literature actually agrees on describing digital technologies as inherently disruptive (Jahangir & Zhiping 2015). Traditional banking institutions that are based on physical interaction with customers that access services exclusively or mainly at a branch, cannot stay untouched by this disruptive technology, as so elegantly described by Clayton Christensen’s work, *The Innovator’s Dilemma* (1997). The banking business is being deregulated, both from within the industry but also by the regulator (e.g. PSD2, Open Banking etc.). The traditional banking paradigm of the *Relationship* bank of the thirteenth century’ Banco di San Giorgio, the *Industrial* bank of the eighteenth century Barclays Bank in the UK or the *Information Technology* Bank of America of the 1950s (Panzarino, 2021; 7, Hildreth 2001) is threatened by digital disruption (Arnold and Jeffery 2016, Kroszner 2015), that, in turn, is dictated by the huge leap of new technology and evolving customer needs, behaviours and high-quality expectations (Pousttchi and Dehnert 2018; Hossain et al. 2021). New entries, fintechs moving into banking such as N26, Revolut, Klarna, Zopa, hybrid bigtech approaches such as Goldman Sachs launching Apple Card and JPMorgan current account for Amazon, or new banks (neo banks) such as Chime, Current, Monzo, Starling, digital banks launched by incumbents such as HelloBank! By BNP Paribas, MoneyYou by ABN Amro, Greenhouse by Wells Fargo, all change the game. As per the majority of practitioners (Broeders and Khanna 2015; Westerman et al. & Cap Gemini 2011) and scholars (Mergel et al 2019; Margiono 2021), without a fully digitized agenda, any incumbent will soon find their offering commoditized and their business model disrupted.

Almost inevitably (Hai et al., 2021; Venkatraman 2017), incumbents have to embark into a digital transformation journey (DTJ) that should see them into a new era. On that journey, bank management will need an understanding for navigating through the different approaches and options, extending beyond the consultancy reports to more systematic empirical evidence (Mergel, 2019) on the stages and the content and context of the DTJ. This paper addresses this issue by identifying three distinct phases that comprise a DTJ and by providing a set of actionable characteristics that each phase have. Bank management and stakeholders can use it for course-plotting.

## Background literature

### Defining digital transformation, digitization, digitalization

Digital transformation has not enjoyed a universal definition among scholars. Vial (2019) in his literature review registers 23 unique definitions. In fact, terms like digitization, digitalization, or digital transformation are used quite often interchangeably in the literature (Mergel, 2019; 10, Reis et al 2018; 412). For the purposes of the paper, we have adapted the definition of Mazzone (2014) the “deliberate and ongoing digital evolution of a company, business model, idea process, or methodology, both strategically and tactically” as the best suited to describe the journey ahead for the incumbent institutions. We will define digitization, as the material process of converting analogue streams of information into digital bits (Brennen and Kreiss 2016), that allows digitized information to be easily stored, transferred, manipulated, and displayed. We will also adapt the definition of digitalization commonly used from Gartner glossary, “Digitalization is the use of digital technologies to change a business model and provide new revenue and value-producing opportunities; it is the process of moving to a digital business”, gartner.com/it-glossary, in Gebauer et al (2020) and Gray and Rumbe (2015).

## Research on digital transformation

The paper does not aim at conducting a systematic literature review on digital transformation and the phases of it. Instead, it draws from the work done by a number of academics. For a more informative literature analysis, we recommend commencing from the work done by Christensen (1997, 2016), Christensen et al. (2015) on digital disruption and some criticism of it (e.g. Danneels 2004), and follow the literature review and analysis papers published by Mergel (2019), Vial (2019), Rosenstand et al. (2018) and then Parida (2019) and Verhoef (2021). An interesting addition is a very recent publication on digital transformation literature review by Reis and Melao (2023), who having employed a meta-review methodology reviewing an extended range of articles, notice that the field started maturing after 2018. Same team, Reis et al. in a previous paper (2018), are mentioning the small representation of digital transformation in academia. These papers help amass the data that have been produced over the past decade on digital transformation, definitions and coherence with business models innovation. Earlier scholars (Markides 2006; Morakanyane et al. 2017) have also contributed.

As far as the banking DTJ is concerned, the concept of which is debated substantially in the fourth chapter of the book of Panzarino and Hatami (2021), we tend to agree with Bouwman et al (2018) who, in their work, conclude that research on DTJ is rather scattered and sometimes lacks depth, as it tends to focus on specific elements rather than the whole entity. We found rather limited academic research on banking digital transformation (Kalsing, Verhoef), while a lot has been posted by grey literature from, virtually, all major professional houses( Selma et al 2021). They also notice that academic research on the subject is still on early stages with no dominating authors and the focus is disseminated on many different areas.

## Research objectives and methodology

### Background information

Digital transformation, as abrupt as it may seem to some industries, it does not come overnight. It has a start, a path and a destination. It constitutes a journey. In describing this journey, practitioners and academics alike (Panzarino et al., 2021; Markides, 2017; Kalsing 2021) provide a number of different roadmap concepts for digital transformation varying on numbers of levels, from three phases of Panzarino and Hatami, (2021, pp. 54–74) and Verhoef, (2021), four paths of digital change (Hertzog, 2019) to the five stages of digital transformation by Saldanha (2019). Venkatraman (2017), employs a 3-stage concept splitting it on digitization, digitalization, and digital transformation, while Costa et al (2015, BBVA Digital Economy Watch), break the process of transformation towards digital banking in three stages of Response, Adaptation and Strategic Positioning. O’Connor et al., (2008) approach it via the radical innovation project life cycle concept, breaking it into discovery (exploration), incubation (experimentation) and acceleration (development). Earlier work acknowledges also a process-phased concept referring to earlier Business Model structures (4I-frarmework, Fraknerberger et all, 2013). What they have in common is that they all agree on a distinct, as well as defined and conscious, metamorphosis path, with its stages and specific milestones as well as measurable metrics.

### Objectives of the research

This paper seeks to evaluate the digital maturity journey (DTJ) of the incumbent banking industry and to ascertain a path. Three research questions are being placed.

- Q1. Is there an incumbent banking industry-specific, traceable and recognizable path for their digital maturity journey?
  This question is motivated by the fact that the banking industry has been undergoing a rapid digital transformation in recent years, and it is important to understand whether there is a clear phased path that banks can follow to achieve digital maturity. We have discussed the fact that literature has provided different approaches to the stages of digital evolution, and we will be aiming at framing an answer for the banking institutions.
- Q2. Can there be an identifiable set of enablers whose maturity defines the phases?
  This question is based on the premise that achieving digital maturity involves developing specific capabilities and using technology in specific ways. By identifying the enablers that define the phases of the DTJ, we can gain a better understanding of the key factors that contribute to digital maturity in the banking industry.
- Q3. Can a set of Progress Tracking Indicators (PTI) be developed for measuring progress made in the DTJ?
  This question is important because it is essential to track progress made in the digital transformation of banks and to understand whether they are moving in the right direction. By developing a set of tracking indicators, we can better assess the progress and success of the DTJ and identify areas for improvement, providing, at the same time a useful checklist tool to practitioners.

### Data gathering

The authors, applying their expertise on the field, employed three different sources of data for their research. The first source of information was the numerous published papers of grey literature by all established strategy and IT consultancies (namely, Accenture, MIT Center for Digital Business & Cap Gemini Consulting, BCG, McKinsey & Company, PWC). The references represent the reviewed sources, acknowledging that these being no scientific editions have an inevitable degree of repetition, generalization and popularization. Nevertheless, the sheer volume and attention put into the overall subject of the digital transformation and its enablers, demonstrates the relevance it has for the industry and provides an excellent source of practical knowledge. The second source of data came from the work previously done by scholars on the subject of banking digital transformation and its measurements. Quite a limited field yet, the papers that helped us build a better understanding of the scope are presented in the relevant literature support chapter, as well as in the rest of the article. Lastly, we draw also conclusions from a case study research (Yin 2013) conducted on the Greek systemic banking industry. The named research has been presented in full detail in the paper published by the authors titled, “Digital transformation journey for incumbent banks: The case of the Greek financial institutions” (submitted). The exercise remained qualitative and all relevant elements and descriptors (case, bounding system, context, in depth, study, selection, evidence, design) as presented in Table 1, [36] of Harrison et al. on their *Case Study Research: Foundations and Methodological Orientations* (2017) were observed.

**Table 1:** Adaptation phase “Toe in the water”—key enablers with their basic features and descriptions and the corresponding tracking indicators

Enablers	Features of the enabler	Description	Progress tracking indicators
Strategy and organization track	Turn towards digitization of Business	Back-end processes automated across some channels. Repetitive internal actions digitized (invoicing, general ledger fulfilment, contracts, retail customer applications)	% Processes/products/documents digitized vs physical
		Redesign and digitize Customer front end	#, % Customers channelled via e channels for application vs physical/hard copy
		Redesign of key processes with digital focus Roadmap formation to front office/customer facing staff	# Steps/stages reduced per process
	Initial Strategic Alignment and Prioritization for use of modern technology and methodology	Strategic projects selection and championing	Internal assessment (scale of 1–5) for project selection based on predefined features such as impact, expandability, simplification)
		Introduction of new, low cost technology for quick wins	# FTE reduction, %budget impact
		Streamline IT attention and project demand towards Digital developments	% Internal projects with digital scope over total annual plan of IT projects
People and culture track	Management commitment on digitisation and change, overcoming	Creation of distinct Digital Unit department for introducing concept into the rest of the organization	% Response to internal digital job openings
	Embracing of New Digital Age	Change delivery to basic journeys by moving into digitized environment	#Replaced processes and procedures with % reduction of time spend
	Digitized work environment	Staff has access to remote desktop, basic company intranet, some dashboards/Business Intelligence capabilities access, on line meetings, e-learning seminars	% Working time spend on new e-work environments
			Internal Survey on satisfaction
			% Adaptation of new ways by staff, FTE reduction
Technology and innovation track	Business process redesigning-first stages	Internal review, identification and replacement of older approaches (waterfall, focus groups, etc.) with new methodologies. Isolated introductions	# New technologies introduced, % of budget, # of innovations introduced
			# Lean initiatives at Branches/front office
	In house build innovation, experimentation	Limited Business process outsourcing, focus on building and exploiting in house technical and innovation capabilities	# Leads to opportunities, Time to market
			# Of new propositions, % Success/failure rate of new entrants
Value proposition	Introduction of remote access/banking Online/mobile presence and some capabilities	Customer on boarding analogue and digital, e-banking introduction, e–m banking for informative purposes	#Monthly Active Users (MAU)
		Advanced CRM environment	Net Promoter Score (NPS)—or other similar Customer satisfaction measurement
	Introduction of digital retail sales channels	Simple retail product (card, current account) available also in digital channels	Innovation and CX improvement
		On line applications and on boarding and access to data	#Applicants via the channels, % of abandoned

*Source*: own adaptation from literature review

## Results

### Phases of transformational life cycle

The paper examines the distinct phases of preparation, journey, and destination, and the key characteristics and actions taken in each phase. The DTJ is not strictly defined by timelines, but rather by conceptual distinctions, making it important to identify the key features of each phase. As stated, in this paper, we focus on three phases: Adapt, Grow, and Transform that we will examine separately. Understanding the characteristics of each phase is essential for banking institutions seeking to achieve digital transformation and enhance their digital capabilities. The phases are conceptually split rather that distinctly defined and they do not have a strict boundary on timelines, so, providing a very precise cut-off between the above groups/periods and the assignment of individual morphological elements to these periods was not a goal of this research.

### Enablers

Digital transformation is multidisciplinary almost by definition, encompassing strategy, organization, information technology, supply chains and culture as well as “a host of newly defined roles for existing agents” as Verhoef names customers, partners, competitors (2021). He defines some strategic imperatives of digital transformation, i.e. identifies a set of key enablers that are necessary for any digital transformation to go ahead successfully:—Digital Resources—Organizational Structure Growth Strategy—Metrics and Goals (Verhoef et al 2021). Loonam et al (2018;) on a similar approach, stress that management needs to acknowledge the critical perspectives of strategic‐centric, customer‐ centric, organizational‐centric, and technology‐centric views in order to deliver digital transformations.

**Fig. 1:**
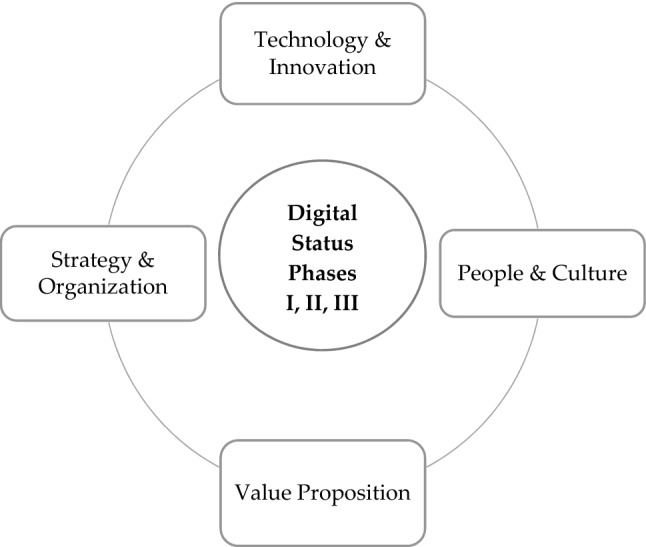
The key enablers to the digital transformation journey

It is the paper’s view that Strategy, People, Technology but also Value Proposition formulate, in one conceptual framework (Fig. 1), the territories that include the facilitators of change that, once mobilized, can help a banking institution move across the stages. They are the contingency factors that can trigger, enable, and may even hinder the digital change, all interconnected, all contributing to every phase. We will examine their contributions per stage, later on. Figure 1 provides a schematic overview of the structure.

#### Strategy and organization

Strategy and Organizational structure are the enablers that will configure the context within which transformation will take place. The approach for what needs to be done should start from a higher perspective on strategic positioning over the long term, it cannot be a stand-alone reaction to external factors or the technology, but it should form part of overall mission and company assessment (Kane et al. 2015; Allchin et al. 2020). Aligning operating model framework and design models with clear strategies is not a characteristic of digital transformation only, but a general management imperative (Campbell and Gutierrez 2021, and, earlier, Grundy 1995; 65–89, Porter 1980). Strategy setting in that context should follow the phases of the journey. Step 1 Diagnostics: this is the more inward-looking stage where each bank needs to do their own omphaloscopy Step 2 Direction: where do the winds blow, where do we stand, where do we want to go, how we do that. Key objective is to read the future customer preferences (Choi et al 2022), the regulatory environment, the technological advancements, the new and existing competitors’ behaviours and direction. Step 3 Transformation program: debate, decision and design of the one or multiple business models that will drive the organization into the new era (Reichert et al., BCG publications, 2022).

#### People and culture

Personnel theories emphasize the importance of getting everybody’s engagement and commitment. Embracing of the Digital Age will be a top down exercise and leadership should make sure (Wide, 2020) that such responsibility is not left to the IT specialists or external advisors/consultants (Selimovits, 2021). If the latter happens, any effort is no longer related to the whole organization but to the individual efforts of specialized departments. The whole team should embrace digital vision and change of culture (Henderikx and Stoffers 2022). Paying attention to the role of senior management in digital transformation is also important. The Chief Digital Officer (CDO) needs to ensure that the transformation process can be orchestrated smoothly. Singh and Hess (2017) and Margiono (2021) state that the CDO plays a different role from other C-level officers because he is responsible for the overall digital initiative and linking the IT and business functions. Training days for the top management, appointment of CDO in the top layer, sponsor of transformation at every major part of the bank, making the top executive team permanent part of the whole change, new digital roles, upskill trainings should also be part of the culture. Empowering all staff through New WoW—New Ways of Working-, remote access and fully digitized environments, will ensure cooperation and smoother transitions. As with all changes, there will be resistance to change efforts. Appropriate goal setting and critical communication can be the determinants on easing the transition (Akan et al. 2016).

#### Technology and innovation

New technologies are disrupting the traditional ways of management (Venkatraman 2017) and are subjecting all enterprises to a number of opportunities as well as challenges both internally and externally, providing a revamped opportunity for revitalization, transforming the very core of the business model as well as the wider environment that the company operates (Barrett et al. 2015). Technology, at its newest forms, is coming, with credit risk assessed by artificial intelligence, customer service covered by robotics, ledgers service back office, contracts serviced via blockchain and all core systems cloud-based (Harris and Wonglimpiyarat 2019). An earlier empirical study, with a strong focus on end-users inside the IT within banks in the German banking industry by Schmidt et al. (2016), revealed that inside experts consider the banks not ready for the last phase. If anything, banks still have to deal with a low degree of integration of their IT systems, not enough optimization of business processes, non-realized automation potentials and a lack of end-user training (Schmidt et al 2017). The alignment of IT strategies with other strategies has remained a difficult and controversial endeavour with further evidence needed as to how this alignment can be conducted in practice—not only related to IT strategies, but also from an organizational perspective (Matt et al 2015). Innovation leads also to open economies, opportunities for platform creations and ecosystems (De Reuver et al 2018) and banks need to embrace it. In a very recent publication, Zhang et al (2023) identify “two innovation types that are conducive to the digital transformation of incumbent firms: process-reconstruction innovation and product-renewal innovation”. Financial institutions, although not included in their research, are matching that pattern.

#### Value offering

What will now be changing dramatically, as the journey moves to its destination, is the reinvention by incumbents of the customer experience (CX). The new customer wants the best experience, in chunk bites and they want it now. Incumbents have to respond. The process begins with bringing in data and analytics-based insights about what really matters to customers and how best to deliver it to them, (Mckinsey by Diaz et al., 2017 and Olanrewaju 2014). It is not easy for many companies, especially those who have not mastered the full concept of digitalization and are still focusing their attention only on the front face of the activity to see it as a complete journey that cuts across multiple functions and channels. Companies must explicitly tie the reinvented CX to their operations (King 2019). If they focus only on the front-end experience and do not change the back-end operations that support it, the new experience is unlikely to be sustainable as per Mckinsey by Diaz et al. (ibid). Changes will be needed in both underlying processes and the way employees work.

### Progress tracking indicators (PTI)

We treat metrics as the perimeter, the connecting points that help managers measure their progress and signify their passing from one phase to the other. Firms tend to employ success metrics related to reaching specific market targets and financial performance. These are the “Key Performance Indicators”, they have been used across industries for a long period (Kaplan and Norton 1996) and the use and practicality of these traditional indicators that ultimately direct to the bottom line such as cost, revenue, ROI is not questioned. The innovation management literature has also employed the terms, aiming at measuring innovation process that is not strictly financially translated (O, Connor et al., 2008), while a similar method employed is the identification of the Key Success Factors (Shah and Siddiqui 2006). As Christensen et al. (2008) argue, the key performance indicators that are to be used to measure success and progress for innovation, cannot rely on traditional financial measuring tools, as these can mislead management into comparing own innovation with the do-nothing option, missing competitors’ effects on longer term profitability of the company. There is need also of proof points that support overall narrative and progress of the specific initiatives (Allchin et al. 2020), moving away from monetary terms. That is not to say that money translated targets should be abolished in transformational projects. The average transformation program that is announced in the wider financial world demands a spending of about 5% of their annual revenue of the organization, spending that includes investment attributed to a host of changes including IT, maintenance, regulatory and compliance, etc. (Allchin et al. 2020). Management should feel that this is money well spent, thus it should be measured.

We propose therefore an adaptation of the approach of Kristiansen and Ritala (2018) who argue that large firms’ radical innovation should be measured by distinct monitoring mechanisms and we will be introducing, at each phase of the transformational journey, a list of primary tracking indicators matching each key enabler, aiming at helping management design the projects and follow-up progress in a more rounded way. In the deployment of the three phases and their corresponding tables, we have focused on the performance trackers that match strategy, people technology and value creation, excluding traditionally established KPIs that, we feel, research and practice have already mastered. These should be used as assessment criteria for the bank to place itself in the ranking vs competitors and self-vision, Grebe et al. (BCG, 2021), Kaufman et al. (2020), Wodzicki et al. (2020). Useful inputs to the search came for professional publications (Digital Directions Team 2022; Choi 2022; Mallick 2020; Schrage et al 2022).

### Phases of the journey

#### Phase I: Adaptation phase “Toe in the water”

The first phase, describes the adaptation to the digital world, which is identified with incumbents looking at aligning digital technology with strategic priorities, mostly translated into improving their front-line offering, without really going into the specifics of the digital concept. It is the time that it is becoming clear that digital models are being embraced across all industry sectors (Weinstein, 2020). Rappa (2010) reports nine types (categories) and more than 40 sub-categories where online models fit well with the paradigm of the relevant industries, including banking & finance. Products themselves are still channelled through their old traditional way and they are designed and manufactured to service only or mainly the existing known channels i.e. branches, while electronic banking is still seen as novelty and complementary (Daniel and Storey 1997). The cost of introducing new technology at this phase is relatively smaller because it is easy to pick up the products, processes and channels that can be digitized with lesser resources, being in the periphery of the core proposition. Even more so, sales at that stage can benefit hugely as long as the institution understands that offering an online card or opening an account is far cheaper (the average transaction cost of branches is approximately 40 times higher than that of online banking, CEB Tower Group 2013), more efficient and more convenient for everybody. At these stage IT legacies remain as such, legacies and challenges. With the introduction of a completely new set of functionalities, modules and applications that now intercut with core banking, back-office processing systems are overloaded. At the same time, customers are accessing far more often their information data due to the early adaptation of online banking, increasing the pressure, resulting in disruptions, downtimes and a lot more problematic maintenance of the old systems of the bank (Panzarino). The digital potential is being understood for simple transactional interactions and digitization of back end processes but support to structural changes is still relatively low in the bank (Selimovits, 2021), cautiously avoiding challenge of status quo. Staff reductions are becoming a reality, creating a negative morale connotation for such changes. Management starts committing the organization to the new digital age.

Introduction of innovation is usually first appointed to in-house sources, mostly by the IT departments and known external IT suppliers. It is the safest approach, it starts cheaper and faster and with the added advantage of originality, since no one else would have access to such internal developments. Information Technology banks, i.e. the prevailing banking model of incumbents, having built a culture of dominance for themselves, would find it hard at that point to open the doors for cooperation with outsiders. Unknown costs that can easily escalate, scarce in-house talent, politics, will soon erode the beliefs in that approach. Most digital initiatives are an isolated approach.

Table 1outlines the key enablers and features required for an organization to turn towards digitization of business. The first track, Strategy & Organization, focuses on automating back-end processes, redesigning the customer front end, and streamlining IT attention towards digital developments. Key PTIs include the percentage of processes/products/documents digitized versus physical, the number of steps/stages reduced per process, and the percentage of internal projects with digital scope over the total annual plan of IT projects. The second track, People & Culture, highlights the importance of management commitment, creating a distinct digital unit, and embracing the new digital age. PTIs include the percentage of response to internal digital job openings, the percentage of staff adaptation of new ways, and the percentage of time spent working in new e-work environments. The third track, Technology & Innovation, focuses on business process redesigning, in-house innovation and experimentation, and introducing lean initiatives at branches/front office. PTIs include the number of new technologies and innovations introduced, the percentage of budget allocated, and the time to market for new propositions. The final track, Value Proposition, highlights the introduction of remote access/banking online/mobile presence, an advanced CRM environment, and digital retail sales channels. PTIs include the number of monthly active users, the net promoter score, and the number of applicants via digital channels.

#### Phase II: Growing phase “Free style swimming”

On the second phase, the pace of transformation is picking up by entering digitalization. The new business model options are coming into the scene. Abbott (2021) provides four strategies for a banking business model (that could be stand-alone or a mix): Sell only own products—the current incumbent approach-, build a distribution-driven ecosystem, sell own capability as a service or create new propositions through non-linear business models. Incumbents could set themselves these questions: Become an infrastructure provider (a shrinking but low costs defending action that requires the bank to provide financial services via other distributors with limited customer ownership) or a lifecycle central point (an expanding option with platform build up and extra services requirements). All scenarios will require next generation capabilities set up (back office, UX, AI, networks, staff skills). Paradoxically, this is the situation where banks may actually increase their costs having to maintain their very old IT legacy and build the new approach and the new systems. The management of the organization should be having a Digital Vision that shares with all. Digital Executive Board operational progress tracking fora, C-suite level appointment, creation of new department that advocate the digital adoption such as Centre of Excellence, as well as new job profile categories should be helping change the culture throughout the organization.

A fintech strategy is supposed to solve a problem. Instead of spending years to develop internally a new scorecard (and repeat), it is easier to work with a fintech start up to develop machine-learning scorecards that keep evolving. Such specialized companies have the capability “to provide banking services in 22 out of 36 Bank domains” as illustrated in the work of Hanafizadeh and Amin (2022). As per Derek White, Barclays' chief design and digital officer, Barclays has estimated that it is five times cheaper and three times faster for the bank to work with tech companies to find solutions to its cybersecurity, CX or big-data problems than trying to find a solution for themselves (Crowe 2015). Many new entrants are now coming into the ecosystem. The choice is not clear nor unlimited. As a worldwide survey by Deloitte presented in FinForum Greece, 2021, offered services of fintech and other new players (novobanks) are not mature enough as yet, as 80% of digital champions come from traditional banks and just 20% are challengers.

At this stage, market drives reduction of branch footprint, as the management starts to realize that physical branches may no longer be needed. The positive correlation between physical and digital is a quest. So is the role of the personnel. As evidence of the research by Kaur et al (2021), branch staff he's pivotal for communicating the digital benefits to clientele. Banks will be removing some traditional staff roles, back-office data entry, branch tellers, old legacy operations staff, call centre agents and will be adding some brand-new roles like business analysts and big data interpreters, fintech specialists, relationship managers for incubator labs, tech advisors for hackathon organizations, digital product and digital sales, social media content managers (King 2019). Likewise, leaders will need to be equipped with appropriate digital skills, contributing to the optimal development of the team (Hai Thanh et al 2021). It is only natural that not all will go smoothly. The role of Chief Digital Officer is one that will concentrate all enthusiasm, tension and failures of that digital revolution (Wide, 2020). No wonder that the average manager in the role has a much smaller occupancy time expectancy compared to the other C-level peers, (Papathomas and Konteos 2022). Agile and Design Thinking methods are fuelling innovation adaptation (Ghezzi and Cavallo 2020).

The value proposition has to offer customer access to internally produced propositions but also provide access to outside offering. The relevant technology, Open Banking, APIs (Zachariadis and Ozcan 2017) is there, as well as the regulatory environment e.g. PSD2. The use of online sharing platforms in other industries has substantially changed the perception of the idea. Uber increased the efficiency of underutilized assets and lowered the consumption prices of the services for the customer, while E-commerce platforms, such as Amazon Marketplace, have greatly reduced transaction costs for many small and medium-sized enterprises (SMEs) to sell products across states and borders, Li et al. (2018). Now the bank does (or should) think of concepts of fully developed platforms and ecosystems with its offering in the middle and with key decisions that will alter its DNA. The bank should normally by now have optimized front-end channels and should have decided on a business path forward. The first-generation products and services made for Digital First environment should now be coming into the market. Yet, now the first enthusiasm is passing and problems arise. Service should be of similar experience to every option and every channel used by the end user so that customer experience CX is common and uninterrupted. Omnichannel delivery is becoming a necessary model adaptation (Zhou et al. 2020), where the sale experience and service are seamless and meld the advantages of physical stores with the information-rich experience of online accessing, irrespective of the device, Lazaris and Vrechopoulos, (2014) and Saprikis et al. (2022). The branch remains centre—although COVID-19 has done a lot to weaken that notion (see Bechlioulis and Karamanis 2022 for example)—with the belief that all customers can use it, not everybody has access, understanding or interest in specific channels for example e-mobile (customer “branch skills” vs customer “digital skills”, Panzarino, pp 59).

Table 2highlights the key enablers and features required for an organization to turn towards digitalization of business. The first track, Strategy & Organization, focuses on automating back-end processes across all channels, reducing paper usage, and turning non-customer facing units digital. progress tracking indicators include the number of processes that become digitized, the percentage of digitization within processes, the internal steps/stages reduced per process, and the real-time view of transactions. The second track, People & Culture, emphasizes the importance of ensuring a bank-wide focus on top management's digital vision, creating a digital transformation office, and promoting the need to turn digital throughout the organization. PTIs include the percentage of response to internal digital job openings, the percentage of working time spent on new e-work environments, and the number of initiatives produced. The third track, Technology & Innovation, focuses on adopting new methodologies, creating an ecosystem and integration using fintechs, and applying a mobile-first approach. PTIs include the number of new technologies introduced, the feedback received, and the budget allocated. The final track, Value Proposition, highlights the mobile-first approach, an omnichannel fully operational model, and digital sales. PTIs include the percentage of customers channelled via new channels, the net promoter score, and the percentage of sales via e-channels. By implementing these key enablers and features, an organization can successfully turn towards digitalization of business, resulting in innovation and improved customer experience.

**Table 2:** Growing phase “Free style swimming”—key enablers with their basic features and descriptions and the corresponding tracking indicators

Enablers	Features of the enabler	Description	Progress tracking indicators
Strategy and organization track	Turn towards digitalization of Business	Back-end processes automated across all channels	# Processes that become digitized, % of digitization within processes
			% Paper usage/printer consumptions = > Paperless in X years
		Non-customer facing Unites (Operations, Support Functions, HR, Risk) turn digital	# Internal steps/stages reduced per process, €, FTE reduction
		Use of smart process technology to create data/Big Data Analytics and alignment with digital	Real time view of trxs (Y/N)
			# Users of Big Data, Reporting productivity/efficiency feedback
	Business Strategy driven by Digital Vision	From running uncoordinated efforts within siloes to launching an integrated operational-improvement program organized around journeys	Top management feedback
			Internal Survey measuring alignment/scorecard
		Budget, Targets, resources commitment for digital transformation	# Initiatives with digital scope over total number of engaged activities
		R&D investment directed towards digital initiatives	% R&D investment on digital projects vs all
	Change delivery framework establishment	Digital Factory Creation to work as the centre for digital excellence and diaspora	# Initiatives initiated, # Initiatives delivered
People and culture track	Ensure bank-wide focus on top management Digital Vision	Digital Executive Board creation, operational progress tracking fora	#Meetings, events, projects sponsored by Board (self-assessment)
		C-suite level appointment for digital overview	Digital adoption target setting to Business Units of the bank (scorecard)
		Set up Digital transformation Office Centre of Excellence, or CoE, (centralized place where best practices are formulated based on knowledge and data from a company’s experience)	Digital Content creation and optimization (1–5 ranking)
			#Initiatives produced
	Digital adoption and change of culture throughout the organization	New job profiles, such as Big Data analyst, Innovation managers, fintech specialist, digital products and sales managers	% Response to internal digital job openings
			# Jobs in Digital
		Digital upskills training for staff, Active internal promotion of need to turn digital	% Working time spend on new e-work environments
			# Trainings/staff participation
	Digitalized work environment	Self-service smart platform	Internal Survey on satisfaction
		Cloud based access, mobile access, on line doc sharing and meetings, gamification in digital learning, individual dashboards	% Adaptation of new ways by staff
Technology and innovation track	Fail fast culture/Agile and Design Thinking project approach	Apply new methodologies Customer Journey redesign, Agile, Design Thinking, Lean/Six Sigma	# New technologies introduced, feedback, # staff trained
			% Budget allocated
			Leads to opportunities, Cost Savings
	Ecosystem creation and integration, Use of Fintechs	API, Open banking platform, PSD2-enambed capabilities	# API hits, platform # Active and Repetitive Users
		Hackathons and fintech trails and cooperation’s	# Innovations introduced
		Open and flexible system architecture with Data away from old legacy systems	% Usage by staff, # data downloaded, # reporting/self-usage
Value Proposition	Mobile first approach	m-banking for individuals and businesses	#, % Customers channelled via the new channel, % customer take up of e-banking features
			Net Promoter Score (NPS) -or other similar Customer satisfaction measurement
	Omnichannel fully operational	e-signature, Biometric appliances, contactless interactions, digital issuance of products and services, electronic wallets, Account aggregation	#, Quality assessment from social medias’ mentions
			Innovation and CX improvement, # of mix journeys
	Digital Sales	Digital Sales are add-ons to branches	% Sale via e-channels, # of products passed via e-channels

*Source*: own adaptation from literature review

#### Phase III: Transformation phase “Deep Dive”

The third phase is where revolution, not evolution, kicks in. At this stage, all market forces, customers, regulators, banks, challengers are becoming more adaptable and knowledgeable of the new paradigm and, external to the banking business, players are coming for participation. Banks, at that transformational stage, can remove 20 to 25 per cent of their cost base by leveraging this digital shift to transform how they process and service (Olanrewaju 2014). This is the (future) state where survivors are making their point while late adaptation or failed efforts are taking their tolls both on challenger players and incumbents. This is by far the hardest state on incumbents’ effort to disrupt innovatively themselves and move into a new Business Model (BM). As Markides defines it, (2006), Business-model innovation is the discovery of a fundamentally different business model in an existing business. EasyJet, and Dell compete in their respective industries in substantially different ways from their competitors, such as British Airways, and HP; Amazon sets different standards from a chain bookstore. A disruption will have to be much more than just a new strategy for expansion (Markides, ibid), it will have to aim for a whole lot bigger and better marketplace, moving from a well-established and familiar mondus operandi to a new operating model (Panzarino; 55).

The bank has by now transferred most of its in-house branch capabilities into its external channels and it is ready to offer and possibly already does, digital only products. Barclays and the other High Street Banks in the UK have removed totally card and loan applications from branches. This is a full transformation state, a stage not without its perils or hard decision-making. Incumbents rarely create new digital business models; they are more likely to use digital technologies to extend or improve their existing activities in an evolutionary manner (Foss and Saebi 2017; Volberda et al. 2021; Warner and Wager, 2019). It is rather difficult to fundamentally alter a business model so that it reflects the changes brought about both by new digital technologies and changing customer needs and competitive dynamics (Chesbrough 2010). Research on the adoption of new technologies indicates that cognitive barriers affect the process; managers may not fully understand the structural impediments to change, and dominant logics within the firm may prevent people from adopting new ways of thinking (Chesbrough 2010; Chesbrough and Rosenbloom 2002). In fact, as the process of adopting digital technology is one of experimentation, trial-and-error learning, and discovery (see, for example, Frankenberger et al. 2013; McGrath 2010), the speed with which new digital business models are envisioned and the direction taken hinge critically on decision-makers’ cognitive make-up, which determines their awareness and understanding of the key issues and the range of possibilities for refashioning the business model (Chesbrough 2010; Sosna et al. 2010). It may be then to no surprise that the first intentions of banking industry, as described by CEOs, are an adaptation of a Phygital model (description at Anker 2021), a “safe bet” in that context.

By now, the bank has built its own in-house team of experts recognizing that the traditional accounting and econometrics degrees have, to a certain extent, to be replaced by coding programming and online marketing degrees. Executives need to address the organizational barriers that may arise during the transformation process, especially cultural inertia. Inertia in this context refers to the resistance that emerges during the transformation process, which could lead to slowing down or stopping the process. Inertia can take the form of employee resistance, which may prevent companies from achieving their digital transformation objectives, Margiono (2020). Many banks’ jobs at that stage will be very different from the nowadays-typical banking profile. Skills for digital development (data scientists, artificial learning engineers and blockchain integrators) will be in high demand but other new set of skills will also be required e.g. product managers for designing the CX and ensuring appropriate on boarding identity, behaviour psychologists to blend and fuse banking experiences with life cycle (King 2019).

On the future form of the customer interaction with banks and banking, things are clear. A survey of North American C-level executives revealed that 84% of their customers wanted individualized experiences, which can lead to an additional 18% in annual revenues. It is not really a choice for the banking sector. They have to find the model that produces no more channels or products but experiences. All coming generations of banks customers will expect to have a fully personalized experience, at the time of their liking, the device of their choice, with the service level constant and predictable. This is well demonstrated on the results of a research conducted by Majumdar and Pujari (2022) in the UAE that concluded “perceived ease and usefulness—and information—were the most influential factors on the usage and adoption of mobile banking apps”. In terms of timing, customer experience setting may be coming late for banks, as the retail CX is fully transformed, and business models are already entering the “now” economy. The FAAMG companies—Facebook, Apple, Amazon, Microsoft and Google (and a host of other names of course) are setting the barriers very high for everybody else by teaching retail customers worldwide what to expect—or demand. Millennials will not settle for two working days to clear a money transfer nor will they wait for the branch opening hours to buy stocks (Saprikis and Avlogiaris 2021). It is the Now Economy for Weinstein (2020), Right Now Economy for Tullman (2015).

Table 3outlines the key enablers and features required for an organization to implement a new business model, focusing on a customer-centred digital manufacturing approach. The first track, Strategy & Organization, highlights the importance of running one or multiple business models simultaneously, introducing intelligent process automation to replace human tasks, and centralizing, consolidating, and homogenizing scattered business lines through the use of digital. Key progress tracking indicators include the percentage spend on R&D, the cost of branch network reduction, and the net promoter score. The second track, People & Culture, emphasizes the importance of creating a digital-first culture, embedding digital skills in all job profiles, and implementing an end-to-end paperless back office. PTIs include the percentage of paper consumption, internal survey on satisfaction, and the percentage of adaptation of new ways by staff. The third track, Technology & Innovation, focuses on implementing next-generation technical capabilities, such as artificial intelligence and machine learning, robotic process automation, and blockchain management of contracts and transactions. PTIs include the metrics on applied systems, lean process redesign, and the percentage reduction cost to serve. The final track, Value Proposition, highlights the importance of providing instant time-to-yes on applications, multi-service offerings, and a holistic digital service. PTIs include the net promoter score, the percentage customer conversion from app to account, and the cross-sales ratio. By implementing these key enablers and features, an organization can successfully implement a customer-centred digital manufacturing approach, resulting in innovation and improved customer experience.

**Table 3:** Transformation phase “Deep dive”—key enablers with their basic features and descriptions and the corresponding tracking indicators

Enablers	Features of the enabler	Description	Progress tracking indicators
Strategy and organization track	Selection of New Business Model	Running one or multiple Business models at the same time e.g. Phygital, Spin n off, Digital only	Top management feedback
			€, # FTE savings, financial metricises
		Introduce Intelligent process automations to replace human tasks	% Spend on R&D, # FTE savings
	Customer centred digital manufacturing	Combine and merge Branches and online banking in a fully interactive way All trxs are available in digital format, Branches have an advisory role	Cash trx over the counter in branches
			# Customers visiting branches/frequency
		Centralization, Consolidation and Homogenization of scatted origination/product/support business lines via use of Digital, i.e. homogenize common functionalities e.g. payments platforms	€ Cost of Branch Network reduction
		Customer journey and value proposition fully end-to-end digital	Net Promoter Score (NPS), Time savings, € cost out
People and culture track	Digital first culture	Dissemination of digital only Board, disbursement of knowledge roles to all other boards	Top management feedback
		Abolishment of ad-hoc digital business unit and absorption of staff to every signal Business unit	Internal Quality Index reports
		Digital skills embedded in all job profiles	Remote working tools, online collaboration, training, data access
		New job roles (Data scientist, Machine learning specials, Blockchain integrator but also Digital storyteller and Identity brokers)	% Of staff with no banking education ( engineers, code programmers)
		End to End Paperless back office	% Paper consumption
	Digital Servicing Platform	Create an advanced Tech and data platform to champion next generation data analytics, facilitate new paradigm	Take up of initiatives, internal feedback
	WoW—new Way of Working	Seamless work from anywhere, mobile all, e-signatures, advanced e-learning (augmented reality), self-service Business Analytics	% Adaptation of new ways by staff
			Internal Survey on satisfaction
			% Working time spend on new e-work environments
Technology and innovation track	Next generation technical capabilities	Artificial Intelligence and Machine Learning to operate Credit Scorecards, AML, investment banking, etc	(new) Cost of running Operating systems
			Metrics on applied Systems (Risk, credit etc.)
		Robotic process automation	Bot metrics (speed/past factum surveys/repetitions)
		Core Banking systems in cloud	(new) Cost of running Operating systems
			Metrics on applied Systems (Risk, credit etc.)
	Transformation: New paradigm, new internal IT ecosystems that ensures agility, flexibility, adaptability	Lean process redesign (Streamline processes and minimize waste)	€, % Reduction cost to serve, Time to market
		Blockchain management of contracts, transactions	Internal Quality Index reports, Adaptation pace
		Predict behaviour capacity	
Value Proposition	(Most)Products available only in digital channels	Instant time-to-yes on application	Net Promoter Score (NPS)
		Multi service offering, with lifestyle advice, based on individual client, fully digitized products and services	% Customer conversion from app to account
		Gamification of customer Experience (CX)	% Customer shift from traditional to remote channels
	Fully customized and personalized service to individual and businesses/Relationship Banking	Instant access to personalized data on status, best products, consulting	%, € Revenue generated via new digital channels, € revenue generated via x-sales
		Build capabilities that provide customization via predictive modelling not access to private data	
	Holistic digital service	One stop shop for banking and surrounding services	# Cross Sales Ratio
		Upgraded robotics and artificial Intelligent guidance for Customer Service	Customer satisfaction surveys, Customer Service abandon/satisfaction rate

*Source*: own adaptation from literature review

## Discussion and limitations

### Theoretical contribution

The objective of this study was to use academic and professional publications and empirical evidence to analyse and synthesize a framework that could provide a sounding board against which bank executives can compare and understand where their institutions stand in the DTJ and how to track it.

The research commenced with three Questions that defined the study, respectively. Each one contributes to academia in their own right. The contribution of Q1 is that it confirms the existence of a theoretical path that can deliver to banking institutions digital maturity status and highlights the characteristics of the journey from early adaption to growth and maturity. Q2 identified and analysed the four enablers of the process, Strategy, Technology, People, and—a, highly underrepresented in academia, element—Value. The study provided insights into the specific capabilities and technologies that banks need to develop and use to achieve digital maturity. Lastly, the theoretical contribution of Q3 is that it helps to track progress made by listing a set of Progress Tracking Indicators that can be used to assess the success of the DTJ and identify areas for improvement. This allows management of banking institutions and consultants to better understand where their institutions stand in the DTJ and make decisions that are more informed.

Overall, the study's theoretical contributions confirm the distinct, traceable, and recognizable metamorphosis path of digital maturity in incumbent banks and provide a practical framework for banks seeking to achieve digital maturity. The study extends the literature by incorporating all these three concepts (Phases, Enablers, PTIs) into one usable framework, in the form of three Tables, which has not previously been combined in a research context. The field is rather underrepresented in the academic literature, which leads us to believe that academia may have misinterpreted the importance of such practical and measurable indexes in a field that is anyway too new to be saturated.

### Managerial contribution

This paper aims at contributing to a growing, contemporary discussion, hopefully assisting in greater collaboration between practitioners and academics. We believe that the paper adds well to the limited research that relates to digital transformation of banking institutions in three key ways: It progresses further the concepts of the phased transformational journey and the dependant key enablers by dwelling deeper on their characteristics. It also presents an inclusive, defined and articulated list of key tracking indicators that are of immediate use as a tool to bank managers and consultants alike for usage on their digital transformation tactical implementation assessment. This understanding is important in order to make necessary changes and investments in infrastructure and talent, and create a culture that supports digital transformation. The three Tables, that represent the accumulation of the article’s theoretical deployment, can be used as part of a handbook for a digital transformation project plan.

## Research limitations

The findings of the paper may lack the empirical galvanization of the findings and the proven applicability of the theoretical framework i.e. tables combining all three concepts on the ground. Digital banking and all its managerial concepts are still at the very early stages of formation. Furthermore, Key metrics tend to have a wide popularity with practitioners, an interest that is not always mirrored in research journals. Further case studies analysis and follow-ups within the academia will help readjust Phases and Indicators or reconfirm their validity. It is suggested that empirical research—a possible survey or interviews among bank managers—is conducted to verify the above. We also consider that the research objectives (Phases, Enablers, PTI) themselves are too wide and they could be researched separately, giving the opportunity for a more thorough analysis.

### Future recommendations

As stated, research needs further insides to articulate better the findings and expand them on a further cross-examination of relevant theories and approaches. There is a number of questions that came along the study and, we believe that, extension of research to these fields, would help advance the relevant knowledge field:

- Are the four reviewed Enablers the key constituents or should there be a wider perspective? Do they have the same weight within each phase?
- How are Enablers interlinked with each other? Is there an order of hierarchy and should there be other enablers?
- What is the pace of transition from one Phase to another? Can institutions progress in the next phase without completing all enablers satisfactorily? Can financial institutions skip phases all together?
